# BAP31 Plays an Essential Role in Mouse B Cell Development via Regulation of BCR Signaling

**DOI:** 10.3390/ijms25094962

**Published:** 2024-05-02

**Authors:** Bo Zhao, Fei An, Zhenzhen Hao, Wanting Zhang, Bing Wang

**Affiliations:** Institute of Biochemistry and Molecular Biology, College of Life and Health Sciences, Northeastern University, Shenyang 110819, China; 1810068@stu.neu.edu.cn (B.Z.); 2201435@stu.neu.edu.cn (F.A.); 1710065@stu.neu.edu.cn (Z.H.); 2110482@stu.neu.edu.cn (W.Z.)

**Keywords:** BAP31, B cell, development, BCR signaling

## Abstract

B cell receptor-associated protein 31 (BAP31) is a transmembrane protein that is widely expressed and primarily located in the endoplasmic reticulum (ER). B cells play a crucial role in the immune system, and BAP31 significantly contributes to the functions of various immune cells. However, the specific role of BAP31 in B lymphocytes development remains unknown. In this study, we utilized a mouse model with BAP31 deleted from B cells to investigate its effects. Our findings reveal a block in early B cell development in the bone marrow and a significant decrease in the number of B cells in peripheral lymphoid organs taken from BAP31 B cell conditional knockout (BAP31-BCKO) mice. B cell receptor (BCR) signaling is crucial for the normal development and differentiation of B lymphocytes. BAP31, an endoplasmic reticulum membrane protein, directly regulates the BCR signaling pathway and was shown to be significantly positively correlated with B cell activation and proliferation. These findings establish BAP31 as a crucial regulator of early B cell development.

## 1. Introduction

B cell receptor-associated protein 31 (BAP31 or BCAP31) is a transmembrane protein that is widely expressed and primarily located in the endoplasmic reticulum (ER) [1]. BAP31 can function as a carrier protein to mediate the transport of proteins from the ER to the Golgi apparatus for processing and maturation [2,3]. BAP31 has been shown to play important roles in various immune cells. Previous research conducted by our team demonstrated the significance of BAP31 in T cell activation through the T cell antigen receptor (TCR) signaling pathway [4]. BAP31 has been shown to influence macrophage polarization by regulating the activation of helper T cells [5]. It has been observed that BAP31 is predominantly associated with mature B cells expressing the antigen receptor membrane IgD, while its association with membrane IgM is relatively weak [6]. BAP31 has been found to impact various functions of macrophages, such as angiogenesis and skin fibrosis, during the wound-healing process through IL-4Rα [7]. Given its significance in diverse immune cells, it is necessary to further understand the role of BAP31 in the development of B cells. 

B cells originate from hematopoietic stem cells (HSCs) and undergo development in the bone marrow before migrating into the bloodstream to reach peripheral lymphoid organs, such as the spleen. Development in the BM progresses involves sequential stages of pro-, pre- and immature B cells, accompanied by rearrangements of heavy- and light-chain immunoglobulin genes [8]. Successfully rearranged heavy chains and alternative light chains combine to form the pre-B cell receptor (pre-BCR), which regulates the transition from pre-B cells to mature B cells. Light chain rearrangement occurs during the pre-B cell stage, where the successfully rearranged light chain binds to the heavy chain to form a functional B cell receptor. B cells with a functional BCR rapidly process into immature B cells and migrate from the BM into the spleen for further maturation [9]. B cell maturation is controlled by the signals from BCR and eventually gives rise to follicular (FO) and marginal zone (MZ) mature B cells through type 1 (T1) and type 2 (T2) transitional B cell stages.

In this study, we employed BAP31 conditional knockout mice to investigate the role of BAP31 in the development of B cells. Our findings indicate that BAP31 is crucial for early B cell differentiation in the bone marrow. Additionally, we demonstrate that BAP31 plays a crucial role in B cell development by decreasing the number of B cell subpopulations present in peripheral lymphoid organs; the knockout of BAP31 repressed B cell antigen/surface receptors and inhibited B cell receptor (BCR) signaling, thereby leading to attenuated BCR signaling and decreased B cell proliferation and activation. 

## 2. Results

### 2.1. Generating a B Cell-Specific Conditional Knockout BAP31 Mouse Model

**Figure 1:**
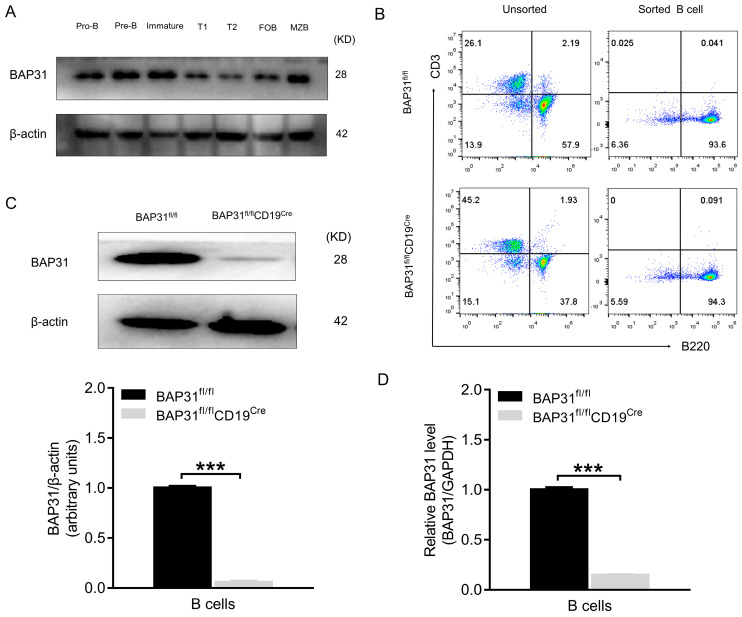
Generating a B cell−specific conditional knockout BAP31 mouse model. (**A**) Expression of BAP31 in the different subpopulations of B cells. Pro−, pre− and immature B cells were sorted from the BM of BAP31^fl/fl^ mice; T1, T2, FOB, and MZB cells were sorted from the spleens of BAP31^fl/fl^ mice. The levels of BAP31 protein expression were measured by means of Western blot, using β−actin as an internal control. (**B**) FACS determined the purity of the B cells from mouse spleens following magnetic sorting. Cells were gated for lymphocytes by size and granularity, and for single cells by doublet exclusion. Single viable lymphocytes were gated by B220. (**C**) Western blotting analysis of BAP31 knockdown efficiency of B cells (*n* = 3). Relative protein expression is expressed as the ratio of the BAP31 to β−actin. (**D**) Real−time PCR demonstrated the knockdown efficiency of BAP31 in mouse B cells (*n* = 3). Relative BAP31 expression was normalized to GAPDH expression. Data are shown as mean ± standard error of the mean (SEM). *** *p* < 0.001 compared with controls using unpaired Student *t*-tests.

We first examined the expression pattern of BAP31 in different subpopulations of B cells. The Western blot results revealed that BAP31 was expressed in B cells at different developmental stages (Figure 1A). Thus, BAP31 is expressed throughout B cell development. To investigate the potential role of BAP31 in B cell development, a B cell-specific conditional knockout mouse model was generated by crossing the BAP31−floxed mouse line BAP31^fl/fl^ with the CD19−Cre line expressing Cre−recombinase under the control of the B cell lineage−specific CD19 gene promoter (Supplementary Figure S1A,B). B cells were isolated from the spleens of both BAP31^fl/fl^ mice and BAP31^fl/fl^CD19^Cre^ mice through magnetic bead sorting. The purity was assessed via flow cytometry; a significant increase in positivity rate was observed in comparison to the pre−sorting cell percentage, surpassing 93%, as depicted in Figure 1B. The extraction of protein and mRNA from mouse B cells was conducted, followed by an examination of BAP31 expression in B cells using Western blotting and real−time PCR techniques, respectively. The outcomes are presented in Figure 1C,D. In comparison to BAP31^fl/fl^ mice, the B lymphocytes of BAP31^fl/fl^CD19^Cre^ mice exhibited a substantial reduction in both protein and mRNA levels of BAP31. The confirmation of a BAP31−specific knockout in B cells was further supported by the analysis of protein and RNA levels, thereby establishing the successful generation of a mouse model. 

### 2.2. BAP31 Deficiency Impairs Early B Cell Development

**Figure 2:**
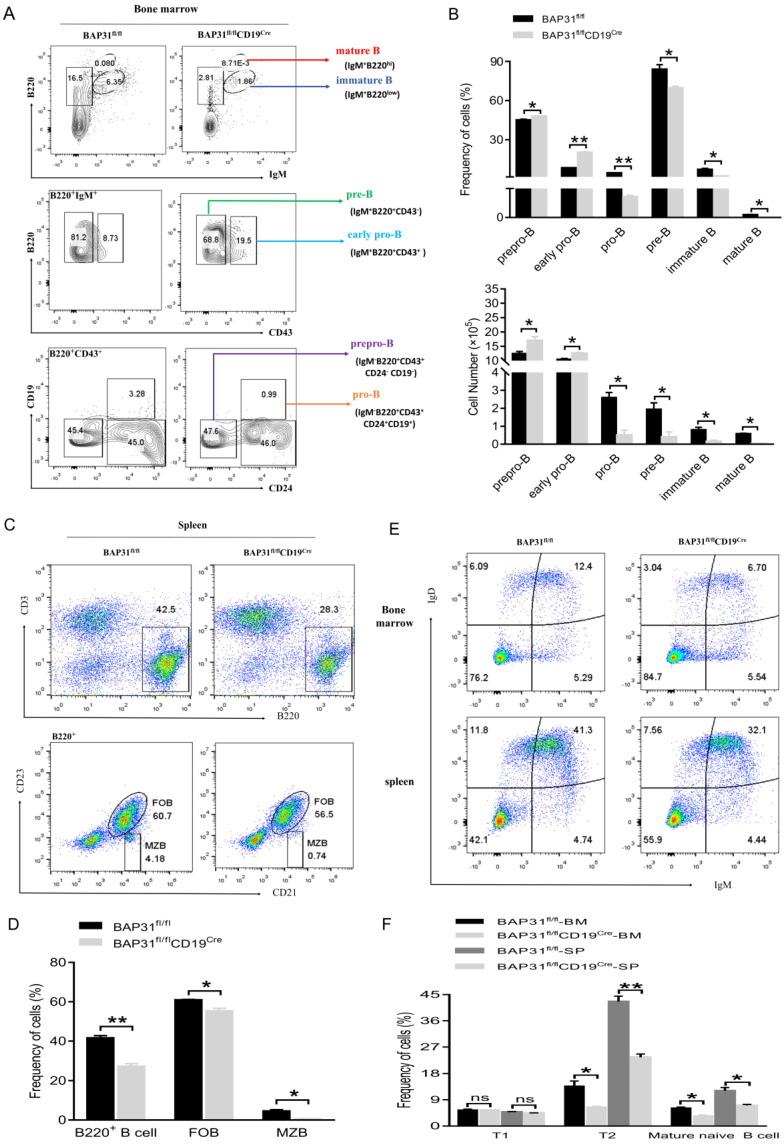
BAP31−BCKO mice exhibit a defect in early B cell development. (**A**) Representative flow cytometry profiles of bone marrow (BM) from BAP31^fl/fl^ mice and BAP31^fl/fl^CD19^Cre^ mice used to identify the percentages and numbers of prepro−B cells (IgM^−^B220^+^CD43^+^CD24^−^CD19^−^), early pro−B cells (IgM^+^B220^+^CD43^+^), pro−B cells (IgM^−^B220^+^CD43^+^CD24^+^CD19^+^), pre−B cells (IgM^+^B220^+^CD43^−^), immature B cells (IgM^+^B220^low^), and mature B cells (IgM^+^B220^hi^) (*n* = 3). Numbers in the plots indicate percentages in each gate. (**B**) Aggregated data for the percentage and total number of different stages of bone marrow B cells. (**C**) B cell subpopulation in spleens of BAP31^fl/fl^ and BAP31^fl/fl^CD19^Cre^ mice determined by means of flow cytometry (*n* = 3). (**D**) Aggregated data for the percentage of subpopulation of splenic B cells. (**E**) FACS analysis of B cell subpopulations in the BM and spleen (*n* = 3). (**F**) Aggregated data for the percentage of subpopulation of BM and splenic B cells. Each group included five mice, and measurements were repeated three times for each mouse; data are shown as mean ± standard error of the mean (SEM). * *p* < 0.05, ** *p* < 0.01 compared with controls using unpaired Student *t*-tests. ns, no significant difference.

We first examined B cell development in the bone marrow (BM) of BAP31^fl/fl^ mice and BAP31^fl/fl^CD19^Cre^ mice using flow cytometry. The findings demonstrated a slight elevation in the percentage and number of prepro−B cells (IgM^−^B220^+^CD43^+^CD24^−^CD19^−^) and early pro-B cells (IgM^+^B220^+^CD43^+^) in the bone marrow of BAP31^fl/fl^CD19^Cre^ mice. Conversely, the percentage and number of pro−B cells (IgM^−^B220^+^CD43^+^CD24^+^CD19^+^), pre−B cells (IgM^+^B220^+^CD43^−^), immature B cells (IgM^+^B220^low^), and mature B cells (IgM^+^B220^hi^) exhibited a significant decline following prepro−B cells and early pro−B cells (Figure 2A,B). These outcomes suggest that the deletion of BAP31 results in a marked decrease in pro−B cells within the bone marrow, subsequently leading to diminished levels of pre−B cells and subsequent stages of B cells. Therefore, BAP31 deficiency impairs early B cell development at the early pro− to pro−B cell transition stage. We next evaluated the impact of BAP31 deletion on B cell maturation. The B220^+^ B cell frequency was reduced in the spleens of BAP31^fl/fl^CD19^Cre^ relative to BAP31^fl/fl^ mice, and the absolute number of B cells was markedly reduced in the spleens of BAP31^fl/fl^CD19^Cre^ relative to BAP31^fl/fl^ mice. Within B cell populations of spleen, the percentages of follicular (FO, CD21^hi^CD23^+^) B cells and marginal zone (MZ, CD21^hi^CD23^lo^) B cells were decreased in BAP31^fl/fl^CD19^Cre^ relative to BAP31^fl/fl^ mice (Figure 2C,D). T1 indicates early transitional B cells (IgM^high^IgD^low^), T2 indicates late transitional B cells (IgM^high^IgD^high^), and mature naïve B cell indicates the B cells (IgM^low^IgD^high^) (Figure 2E,F). Our results show that the population of T2 cells and mature naïve B cell in knockout mice decreased, and there was no significant effect on the population of T1 cells; similar results were observed in the bone marrow and spleen. Therefore, BAP31 plays an important role in B cell maturation. We further examined B cell populations in the lymph node and peripheral blood in both BAP31^fl/fl^ mice and BAP31^fl/fl^CD19^Cre^ mice; as well as a statistically significant change in cell frequencies (Supplementary Figure S1C), our findings reveal a decrease in the cell frequencies of B220^+^B cells in BAP31^fl/fl^CD19^Cre^ mice compared to BAP31^fl/fl^ mice. 

### 2.3. BAP31 Deficiency Impairs BCR-Induced Activation

**Figure 3:**
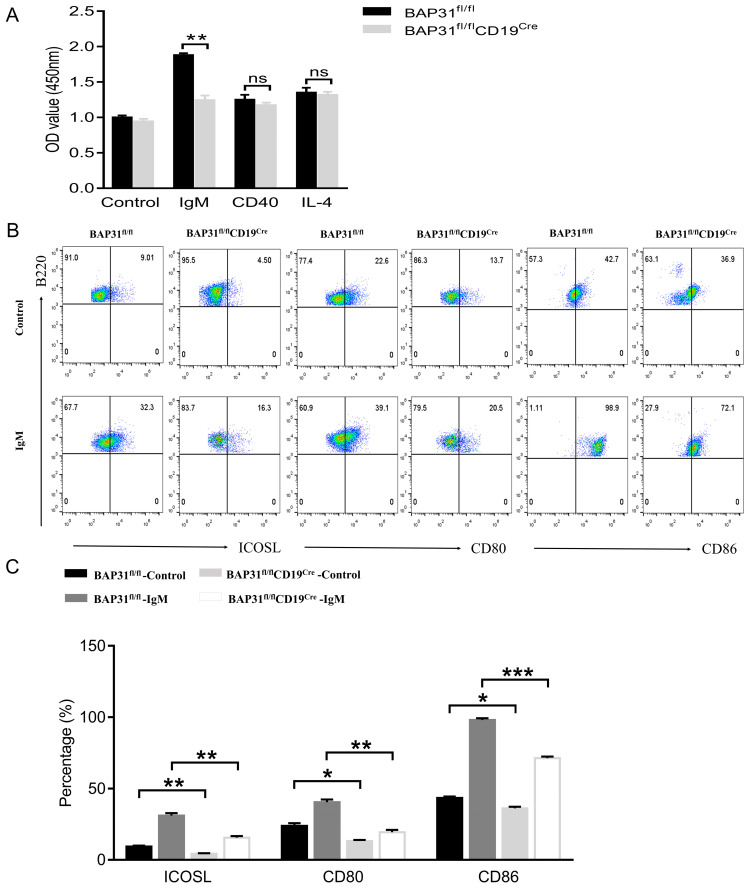
Decreased B cell activation ability in BAP31−BCKO mice. (**A**) Effect of knocking out BAP31 on B cells’ proliferation measured using CCK8 in the different stimulation groups (*n* = 3). (**B**) Flow cytometry used to measure ICOSL, CD80, and CD86 expression in B cells of BAP31^fl/fl^ mice and BAP31^fl/fl^CD19^Cre^ mice from control and IgM stimulation (*n* = 3). (**C**) Aggregated data on the percentage of co−stimulatory molecules of B cells. Data are shown as mean ± standard error of the mean (SEM). * *p* < 0.05, ** *p* < 0.01, *** *p* < 0.001 compared with controls using unpaired Student *t*-tests. ns, no significant difference.

We further analyzed whether BAP31 deficiency affected B cell proliferation, which is a critical event for the number of B cells. IgM, CD40, and IL−4 can stimulate the proliferation of B cells. The proliferation of B220^+^ cells in the spleen under different stimulation conditions was measured by use of a Cell Counting Kit−8 (CCK8) assay. We observed that the proliferation ability of B cells from BAP31−BCKO mice was attenuated compared to BAP31^fl/fl^ mice, and the OD value of the BAP31−BCKO mice was significantly lower than that of BAP31^fl/fl^ mice from the IgM group. In contrast, there was no significant difference between BAP31^fl/fl^ mice and BAP31−BCKO mice in the CD40 and IL−4 groups (Figure 3A). Although both groups showed a decrease, there was no significant difference in the fold increase between the groups. Meanwhile, in vitro experimental culturing did not significantly affect the apoptosis of B cells between BAP31^fl/fl^ mice and BAP31−BCKO mice (Supplementary Figure S2A,B). This proves that IgM could specifically affect the proliferation of B−lymphocytes in BAP31−BCKO mice, which suggests a possible association with anti-IgM−specific BCR signaling pathways. B cells function as antigen−presentation cells, and co−stimulatory molecules play a critical role in antigen presentation [10]. ICOSL belongs to the B7 family of co−stimulatory molecules, and shares sequence similarity with CD80 and CD86 [11]. We also examined the effects of B cell activation under the IgM stimulation condition in B cells from BAP31^fl/fl^ mice and BAP31^fl/fl^CD19^Cre^ mice. Trends toward decreased expression were observed for ICOSL, CD80, and CD86 in BAP31^fl/fl^CD19^Cre^ mice (Figure 3B,C). Results displayed the fold increase value of ICOSL, CD80, and CD86 compared to corresponding controls (Supplementary Figure S2C). The values have no detectable differences between the control and IgM group. This suggests that BAP31 knockout B cells are not actually defective in activation. Rather, due to the reduced BCR signaling in these cells, they are unresponsive to IgM stimulation. Our findings reveal that BAP31 deficiency impairs the BCR−induced activation of B cells.

### 2.4. BAP31 Regulates B Cell Development at Transcriptome Levels

**Figure 4:**
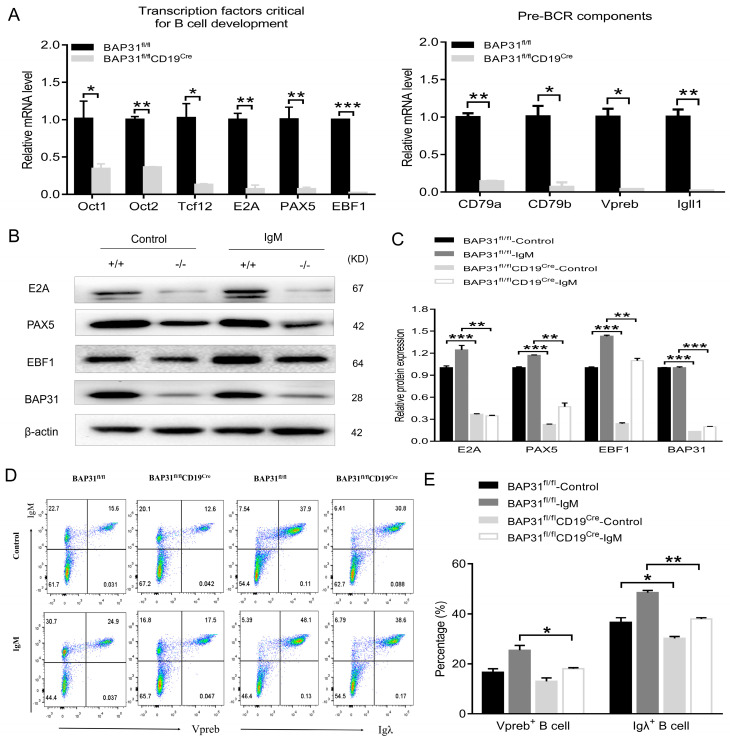
Disruption in early B cell development in BAP31−BCKO mice. (**A**) Real−time PCR validate gene expression of pre−BCR components and transcription factors critical for B cell development from bone marrow of BAP31^fl/fl^ mice and BAP31^fl/fl^CD19^Cre^ mice (*n* = 3). Relative BAP31 expression was normalized to GAPDH expression. (**B**) Western blotting analysis of E2A, PAX5, and EBF1 expression of B cells from BAP31^fl/fl^ mice and BAP31^fl/fl^CD19^Cre^ mice (*n* = 3). +/+ refers to B cells from BAP31^fl/fl^ mice; −/− refers to B cells from BAP31^fl/fl^CD19^Cre^ mice. (**C**) The relative protein expression level was expressed as a ratio of the target protein to β−actin. (**D**) Flow cytometry used to measure Vpreb and Igλ expression in B cells of BAP31^fl/fl^ mice and BAP31^fl/fl^CD19^Cre^ mice from control and IgM stimulation (*n* = 3). (**E**) Aggregated data on the percentage of Vpreb and Igλ expression of B cells. Data are shown as mean ± standard error of the mean (SEM). * *p* < 0.05, ** *p* < 0.01, *** *p* < 0.001 compared with controls using unpaired Student *t*-tests.

Various transcription factors involved in different stages of B cell development, such as E2A, EBF1, and Pax5, work in concert to guide B cell development. Changes in the gene expression of transcription factors crucial for B cell development and pre−BCR components of BAP31^fl/fl^ mice and BAP31^fl/fl^CD19^Cre^ mice bone marrow were measured by means of quantitative real−time PCR (Figure 4A). The results show that the expression levels of pre-BCR components and various transcriptional factors in the bone marrow of BAP31^fl/fl^CD19^Cre^ mice were significantly lower compared to BAP31^fl/fl^ mice. In this study, we analyzed the protein expression level of key transcription factors involved in B cell development and function. We confirmed the expression of E2A, EBF1, and PAX5 in B cells from both BAP31^fl/fl^ mice and BAP31^fl/fl^CD19^Cre^ mice using Western blot analysis. The results of the experiments indicate that the expression of transcription factors for B cell development, E2A, PAX5, and EBF1, were down-regulated in BAP31^fl/fl^CD19^Cre^ mice (Figure 4B,C). We also analyzed the protein expression levels of pre−BCR components. We confirmed the expressions of Vpreb and Igλ in B cells from both BAP31^fl/fl^ mice and BAP31^fl/fl^CD19^Cre^ mice using FACS analysis. The results of the experiments indicate that the expressions of pre−BCR components, Vpreb and Igλ, were down-regulated in BAP31^fl/fl^CD19^Cre^ mice (Figure 4D,E). 

### 2.5. Bioinformatics Analysis of the B Cells from BAP31-BCKO Mice Shows Reduced BCR Signaling

**Figure 5:**
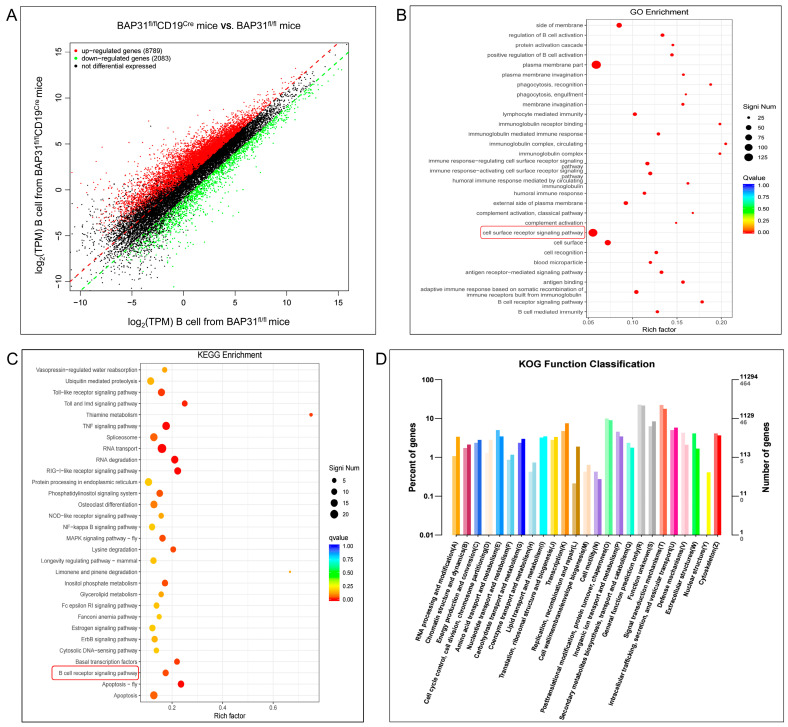
Differences in genes and pathways analyzed using bioinformatics from the BAP31^fl/fl^ mice and BAP31^fl/fl^CD19^Cre^ mice of B cells. (**A**) Scatter plot of transcriptome sequencing results of splenic B cells from the BAP31^fl/fl^ and BAP31^fl/fl^CD19^Cre^ mice. (**B**) Bubble graph of the GO enrichment results of the splenic B cells from BAP31^fl/fl^ mice and BAP31^fl/fl^CD19^Cre^ mice. Each point represents the ratio of genes within the indicated gene ontology term to the total number of differentially expressed genes. The color of the points reflects the adjusted significance level (Q-value), as indicated by the scale. The size of a point reflects the number of genes represented by the point (count), as indicated. (**C**) Enrichment plots of the KEGG pathway analysis with the highest score and lowest *p* value for the enrichment score. (**D**) KOG function classification was performed for the splenic B cells from the BAP31^fl/fl^ and BAP31^fl/fl^CD19^Cre^ mice.

Transcriptome sequencing enables various functional genomic studies of an organism. A Gene Scatter plot has been used to depict differential gene expression, with the up-regulated genes represented by red and the down−regulated genes represented by green, and black represents no significant difference genes(Figure 5A). Gene ontology enrichment analysis (Go analysis) revealed that BAP31 down−expression is strongly correlated with the gene signatures associated with the cell surface receptor signaling pathway, with KEGG enrichment analysis suggesting that BAP31 down-expression may be related to the B cell receptor signaling pathway, as indicated by the red circled portions (Figure 5B,C). Further KOG function classification analysis shows that genes were clustered into 25 functional categories, with genes in the “Signal transduction mechanism” category being the most abundant (Figure 5D).

### 2.6. B Cells from BAP31-BCKO Mice Have Reduced BCR Signaling

**Figure 6:**
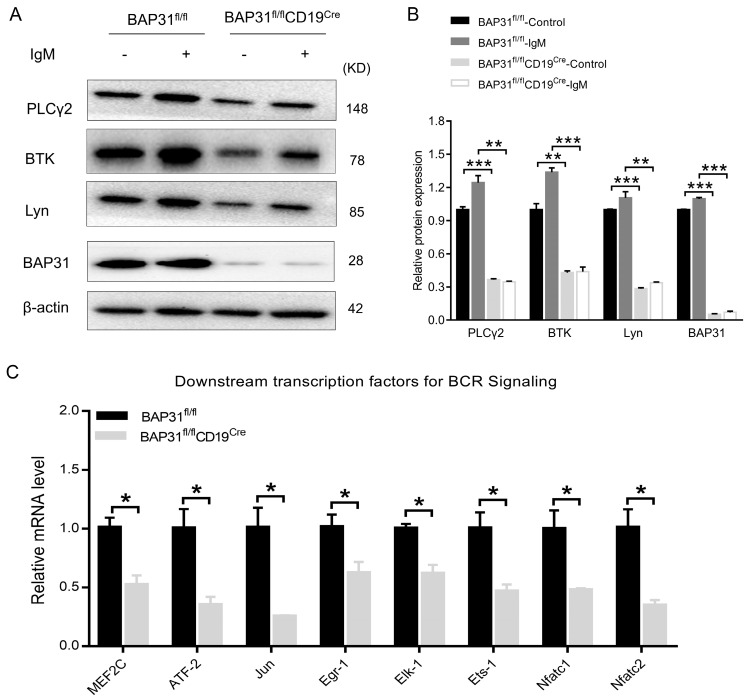
Differences in genes and pathways analyzed from the BAP31^fl/fl^ mice and BAP31^fl/fl^CD19^Cre^ mice. (**A**) Western blotting analysis of PLCγ2, BTK, and Lyn expression in B cells from BAP31^fl/fl^ mice and BAP31^fl/fl^CD19^Cre^ mice (*n* = 3). (**B**) The relative protein expression level was expressed as a ratio of the target protein to β−actin. (**C**) Effect of BAP31 on the downstream transcription factors for the BCR signaling pathways of B cells determined by means of qRT–PCR assay. Data are shown as mean ± standard error of the mean (SEM). * *p* < 0.05, ** *p* < 0.01, and *** *p* < 0.001 compared with controls using unpaired Student *t*-tests.

From the results of bioinformatics analysis, we further analyzed the expression changes of critical BCR pathway members in splenic B cells from both BAP31^fl/fl^ mice and BAP31^fl/fl^CD19^Cre^ mice, including PLCγ2, BTK, and Lyn, via Western blot. The results of the experiments indicate that the expressions of PLCγ2, BTK, and Lyn were down-regulated in BAP31^fl/fl^CD19^Cre^ mice (Figure 6A,B). We also characterized the BCR signaling status of the B cell population in BAP31^fl/fl^CD19^Cre^ mice; the downstream transcription factors of BCR signaling were detected in splenic B cells by means of qRT–PCR. The results show that the relative mRNA levels of key molecules of the BCR signaling pathway were decreased significantly in BAP31^fl/fl^CD19^Cre^ mice compared to those in BAP31^fl/fl^ mice, and the gene expression levels were further reduced in BAP31^fl/fl^CD19^Cre^ mice (Figure 6C). Our results demonstrate that the knockout of BAP31 inhibits B cell receptor (BCR) signaling, thereby leading to attenuated BCR signaling.

## 3. Discussion

Lymphocytes play a crucial role in maintaining the functionality of the immune system. B cells are essential components of the adaptive immune system [12]. BAP31 has been identified as playing critical roles in regulating T cell activation and macrophage polarization, performing important functions in various immune cells [4,5]. In this study, we examined the impact of the conditional knockout of BAP31 on B cell development via the B cell-specific knockout of BAP31 mice models.

The mature naïve B cell repertoire consists of three well−defined populations: B1, B2 (follicular B, FOB), and marginal zone B (MZB) cells [13]. B cells develop in the BM from HSCs. We first assessed the effects of the knockout of BAP31 on B cell progenitor populations in in the bone marrow B cells. Notably, bone marrow B cell subsets, including pro−B cells, pre−B cells, immature B cells, and mature B cells were significantly fewer in BAP31^fl/fl^CD19^Cre^ mice, while there were slightly increased prepro−B cells and early pro−B cells. These results are good indications for BAP31 deficiency impairing early B cell development at the early pro− to pro−B cell transition stage. The knockout of BAP31 also reduces bone marrow and splenic T2 cells and mature naïve B cells. Lower numbers of splenic B220^+^ B cells, marginal zone B, and follicular B cells indicate a possible maturation defect in BAP31-BCKO B cells in the spleen. A lack of BAP31 results in a reduction in B220^+^ cells in peripheral lymphoid organs and is associated with B cell development. Altogether, BAP31 impairs B cell differentiation at the earliest stage of development.

Reductions in B cell populations have been observed in BAP31^fl/fl^CD19^Cre^ mice. To determine the direct effects on B cells, we analyzed B cell proliferation. The co−stimulation of B cells through different co−receptors modulates B cell activation [14]. In our study, we observed a significant decrease in the proliferating capacities of B cells in BAP31^fl/fl^CD19^Cre^ mice; the B cells’ proliferation ability was apparently decreased after anti−IgM treatment. This result reflects a specific involvement of BAP31 in B cell proliferation via IgM/BCR signaling. Molecules involved in B cell activation were further investigated. ICOSL was selected as a marker of B cell activation [15]. CD80 and CD86 are markers of B cell activation, thus suggesting the activated state of the B cells in the different study groups [16]. Therefore, the expressions of distinct molecules associated with B cell activation were evaluated under IgM stimulation conditions. Our results demonstrate significantly decreased levels of activation in BAP31−knockout B cells compared to B cells from BAP31^fl/fl^ mice. Considering the expression of co−stimulatory molecules in control and IgM−treated B cells, the fold increase values were no significant difference between the groups. This suggests that BAP31 knockout B cells are not actually defective in activation. Rather, due to the reduced BCR signaling in BAP31 knockout B cells, they are unresponsive to IgM stimulation. Our results illustrate that BAP31 deficiency impairs the BCR-induced activation of B cells.

In addition, signaling through the B cell antigen receptor (BCR) is required throughout B cell development and peripheral maturation [17]. Stage−specific transcription factors are essential to B cell development [14]. Our RT-qPCR analysis of bone marrow B cells indicates that BAP31 plays an important role in pre-BCR components and transcription factors of B cell development, especially on the transcription factors E2A, PAX5, and EBF1. Moreover, although the reduction in the mRNA level is very prominent, this does not lead to a strong enough effect on the surface expression level, leading to moderate pre−BCR expression on the surface and allowing them to proceed to further differentiation. These results suggest that the knockout of BAP31 may reduce the proliferation and activation abilities of B cells through the regulation of the BCR pathway.

The BCR pathway controls B cell survival, proliferation, and activation, and is a key player in the signaling machinery of B cells [18]. Firstly, KEGG enrichment analysis suggests that BAP31 down−expression may be related to the B cell receptor signaling pathway. Thus, we examined the expressions of three major proteins related to the BCR signaling pathway, according to the results of impaired B cell activation. BTK and PLCγ2 are essential to BCR signaling [19], and Bruton’s tyrosine kinase (BTK) is an essential molecule involved in BCR signaling [20]. Lyn is a tyrosine kinase expressed in B cells that mediates the response from the BCR [21]. We tested the protein expression levels of molecules involved in BCR signaling, including PLCγ2, BTK, and Lyn. Our results show that the expressions of PLCγ2, BTK, and Lyn in the B cells of BAP31^fl/fl^CD19^Cre^ mice were significantly reduced at the protein level. These observations indicate that the knockout of BAP31 inhibited the BCR signaling pathway and is responsible for reducing BCR signaling pathway−associated proteins. Likewise, via the quantitative real−time PCR (qPCR) analysis of downstream transcription factors of BCR signaling pathways, we found a decrease in genes involved in BCR signaling in BAP31^fl/fl^CD19^Cre^ mice relative to BAP31^fl/fl^ mice B cells, coinciding with low levels of protein expression. In the present study, we have demonstrated that the knockdown of BAP31 has the potential to modulate B cell activity not just by down-regulating cell surface antigens associated with B cell activation, but also by reducing the expression of genes involved in B cell proliferation, activation, and differentiation pathways. Nevertheless, further research is required to determine the specific mechanisms of BAP31 that impair B cell development.

In conclusion, the knockout of BAP31 inhibits a few of the key molecules in the BCR signaling pathway and impairs B cell differentiation at the earliest stage of development. BAP31 may serve as a target for new therapies in diseases related to B cell disturbances.

## 4. Materials and Methods

### 4.1. Mouse Models

The mouse strain with fixed alleles encoding the BAP31 gene is a C57BL/6 background, with a loxp locus placed on both sides of the BAP31 gene. Homozygous BAP31^fl/fl^ mice (C57BL/6 background) were intercrossed with CD19−Cre transgenic mice (C57BL/6 background). Cre enzyme-mediated recombination can delete the BAP31 gene sequence of B cells and generate BAP31^fl/fl^CD19^Cre^ animals. The control mice were littermates possessing two loxp−flanked alleles without Cre (BAP31^fl/fl^). Experiments were carried out using 6− to 8−week−old female mice. The mice were maintained under an alternating 12 h light/dark cycle. Animal care and use adhered to the Animal Research Committee guidelines of the Institute of Zoology, the guidelines approved by the Institutional Review Board of the College of Life and Health Science, Northeastern University (Shenyang, China).

### 4.2. B Cell Isolation

Spleens from mice were harvested, and single−cell suspensions were prepared. Single−cell suspensions of lymphocytes were extracted from spleens of mice using Mouse Spleen Lymphocyte Separation solution (Solarbio, Beijing, China). B cells were isolated from murine spleen or bone marrow single-cell suspensions using a MagniSort^®^ Mouse B cell negative selection Enrichment Kit (invitrogen, Waltham, MA, USA). A purity of B cells (B220^+^) of over 90% was detected via FACS analysis.

### 4.3. Flow Cytometry Analysis

Flow cytometry was performed to determine the lymphocyte counts and frequencies of each subpopulation of B cells from mice. Single−cell suspensions from the mouse spleen, inguinal lymph node, peripheral blood and bone marrow were prepared using a 300−mesh nylon screen cell strainer filter. Red blood cells were removed using red blood cell lysis buffer. The cells were resuspended in ice−cold PBS containing 0.1% BSA and then incubated with antibodies on ice for 20 min. The stained cells were washed twice and resuspended in ice-cold PBS containing 0.1% BSA, and then tested on Flow Cytometers (BD LSRFortessa, San Jose, CA, USA) and analyzed with FlowJo software (Version 10, TreeStar, Woodburn, OR, USA).

### 4.4. Cell Apoptosis Analysis

Cells were harvested via centrifugation at 1500 rpm for 5 min and washed with 1 × PBS three times, before being resuspended in binding buffer. Annexin V-FITC reagent (4 μL) and propidium iodide (PI) (8 μL, Beyotime, Shanghai, China) were subsequently added to the samples and gently mixed. Following a 15 min incubation in the dark at room temperature, the stained cells were promptly analyzed using a flow cytometer (BD Biosciences, San Jose, CA, USA).

### 4.5. Abs and Reagents

For flow cytometry, PE-cyanine7-conjugated anti-B220 (clone: RA3-6B2), PE-conjugated anti-IgM (clone: II/41), anti-ICOSL (clone: HK5.3), anti-CD21 (clone: HB50), FITC-conjugated anti-CD43 (clone: eBIoR2/60), anti-CD3 (clone: 145-2C11), CD5 (clone: 53-7.3), anti-IgM (clone: II/41), Percp-cyanine5.5-conjugated anti-CD24 (clone: M1/69), and APC-conjugated anti-CD23 (clone: EBVCS2) were purchased from eBioscience( San Diego, CA, USA). APC-conjugated anti-CD19 (clone: 6D5), anti-Igλ (clone: RML-42), and PE-conjugated anti-Vpreb (clone: R3) were purchased from bioledgend( San Diego CA, USA). PE-conjugated anti-CD40 (clone: RA3-6B2), anti-CD80 (clone: 16-10A1), and anti-CD86 (clone: GL1) were purchased from BD Biosciences(San Jose, CA, USA). For immunoblotting, the primary Abs against PAX5 (catalog no. D7H5X), EBF1 (catalog no. 50752), PLCγ2 (catalog no. 3872T), and Lyn (catalog no. 2796T) were purchased from Cell Signaling Technology( Boston, MA, USA). The primary Ab against BTK was purchased from Signalway Antibody (catalog no. 32321-1). The primary Ab against E2A(catalog no. WL03130) was purchased from Wanleibio( Shenyang, China). The Ab against β-actin(catalog no. 66009-1-Ig) was purchased from Proteintech (Chicago, IL, USA). The Ab against BAP31 was raised in our laboratory as previously described [7]. For cell stimulation, anti-mouse IgM and anti-CD40 were purchased from Invitrogen (catalog no. 31804) and BD Biosciences (catalog no. 550285), respectively. LPS (catalog no. L2630) was purchased from Sigma-Aldrich( St. Louis, MO, USA). IL-4 (catalog no. 214-14) was purchased from Peprotech( Rocky Hill, NJ, USA).

### 4.6. Quantitative Real-Time PCR Analysis

The total RNA from tissues or cells was extracted using RNAiso Plus (Takara, Code no. 9109); cDNA was further synthesized using a HiFiScript cDNA Synthesis Kit for RT-PCR (CWBIO, catalog no. CW2569M). Quantitative real-time PCR (qRT-PCR) was performed with the UltraSYBR Mixture (CWBIO, catalog no. CW0957M) following the manufacturers’ instructions. The primer sequences (Sangon, Shanghai, China) for BAP31, CD79a, CD79b, Vpreb, Igll1, Oct1, Oct2, Tcf12, E2A, Pax5, EBF1, MEF2C, ATF-2, Jun, Egr-1, Elk-1, Ets-1, Nfatc1, Nfatc2, and GAPDH are shown in Table 1.

### 4.7. Western Blotting Analysis

Purified splenic B cells or IgM-activated B cells (treated with 10 mg/mL IgM for 24 h) were stimulated in serum-free RPMI 1640 medium for indicated time periods and then lysed with lysis buffer (8 M urea, 2 M thiourea, 3% SDS, 75 mM DTT, 0.05 M Tris-HCl (pH 6.8), and 0.03% bromophenol blue). Cell lysates were separated using standard 12% SDS-PAGE and analyzed by means of Western blotting using the Abs against EBF1, E2A, PAX5, PLCγ2, BTK, Lyn, BAP31, and β-actin.

### 4.8. RNA Seq Cell Sample Sequencing Preprocessing

The bone marrow B cells were washed twice with pre-cooled PBS, followed by the addition of 1 mL of Trizol reagent to the cells. The reagent was drawn and blown back and forth using a pipette to lyse the cells in the Trizol reagent. The resulting lysate was then transferred to a 1.5 mL centrifuge tube, with the tube mouth sealed using a sealing film. The tube was subsequently frozen in a −80 °C freezer prior to sample transportation. Throughout this process, all steps were performed on ice. For sample transportation, dry ice was used to maintain the frozen state of the samples, which were then sent to Sangon Biotech company( Shanghai, China) for RNA seq sequencing.

### 4.9. Cell Counting Kit-8 (CCK8) Assay

B cell proliferation was measured using a CCK8 assay. B cells were sorted from mouse spleens and resuspended at 1 × 10^7^/mL in PBS. Cells were seeded into a 96-well plate at a density of 5 × 10^3^ cells/well, either unstimulated (incubated in media alone) or stimulated with one of three different immune system stimulants (the presence of anti-IgM, CD40, and IL-4), and cultured for 72 h. BAFF (4 ng/mL) was added to the medium to promote B cell survival in all groups. The viability of cells was determined using Cell Counting Kit-8 systems (Dojindo Laboratories, Tokyo, Japan). After the cells were incubated for an hour at 37 °C, the OD450 value of each well was determined using a spectrophotometer (Bio-Rad, Hercules, CA, USA). The experiments were performed in triplicate.

### 4.10. Statistical Analyses

All statistical analyses were performed using Graph Pad prism 9.0 software (GraphPad Software, San Diego, CA, USA). A statistical comparison was performed using a two-tailed Student’s *t*-test. Symbols indicate statistical significance as follows: * *p* < 0.05, ** *p* < 0.01, and *** *p* < 0.001. Data are expressed as mean and standard error (mean ± SE).

## 5. Conclusions

In conclusion, our findings demonstrate that the knockout of BAP31 regulates the proliferation and activation of B cells through the BCR signaling pathway. Taken together, our findings provide evidence that the knockout of BAP31 plays a significant role in the development of B cells.

## Figures and Tables

**Table 1:** Primers used in this study.

NCBI Reference Sequence	Primer Name	Primer Sequence (5′→3′)
NM_031168.1	BAP31forward	5′-ATGAGTTTGCAGTGGACTACAGTTG-3′
	BAP31 reverse	5′-CTCCTCCTTCTTAGCTGAGGGAC-3′
NM_007655.4	CD79a forward	5′-GACCATGGACGATCTGTTTC-3′
	CD79a reverse	5′-CGTGAAAGGGGTTATTGTTG-3′
NM_001313939.1	CD79b forward	5′-GACAAGGTGCAAAAAGAGGA-3′
	CD79b reverse	5′-GATTCCATGTGGATCAGAGC-3′
NM_016983.1	Vpreb forward	5′-GTGGAGGCATGTTCGGTAGT-3′
	Vpreb reverse	5′-CCAAGTGCAGAGGTGTCTGA-3′
NM_001190325.1	Igll1 forward	5′-CCAGTGGGTCTCATCCCTTA-3′
	Igll1 reverse	5′-AGAAATCCGAGAAGCACGAA-3′
NM_001368808.1	Oct1 forward	5′-TTCAGTGCAGTCAGCCATTC-3′
	Oct1 reverse	5′-GGCTTTGCTGAGGTAGTTGC-3′
NM_001163554.2	Oct2 forward	5′-GGAGCTGGAACAGTTTGCTC-3′
	Oct2 reverse	5′-GATGCTGGTCCTCTTCTTGC-3′
NM_001253862.1	Tcf12 forward	5′-ATTTATTCCCCTGACCACAC-3′
	Tcf12 reverse	5′-GTAGCACATGGATAGCATCA-3′
NM_001164147.2	E2A forward	5′-GATCTACTCCCCGGATCACT-3′
	E2A reverse	5′-GGCATGGTTATGCAAAAGAC-3′
NM_008782.3	Pax5 forward	5′-GGGCTCCTCATACTCCATCA-3′
	Pax5 reverse	5′-CGTCAAGTTGGCTTTCATGT-3′
NM_001290709.1	EBF1 forward	5′-TGCGGAAATCCAACTTCTTC-3′
	EBF1 reverse	5′-GGTTCTTGTCTTGGCCTTCA-3′
NM_001170537.2	MEF2C forward	5′-CACCTACATAACATGCCGCC-3′
	MEF2C reverse	5′-TGGTGGTACGGTCTCTAGGA-3′
NM_001025093.2	ATF-2 forward	5′-TGTAATCACCCAGGCACCAT-3′
	ATF-2 reverse	5′-CTGGTTGAGGAGAGGAAGGG-3′
NM_010591.2	Jun forward	5′-TTCTACGACGATGCCCTCAA-3′
	Jun reverse	5′-CCAGGTTCAAGGTCATGCTC-3′
NM_001348026.2	Bcl-6 forward	5′-TCTCAGTCCCCACAGCATAC-3′
	Bcl-6 reverse	5′-AGAAACGGCAGTCACATTCG-3′
NM_007913.5	Egr-1 forward	5′-AACCCTATGAGCACCTGACC-3′
	Egr-1 reverse	5′-CGTTTGGCTGGGATAACTCG-3′
NM_007922.5	Elk-1 forward	5′-TCCCCACACATACCTTGACC-3′
	Elk-1 reverse	5′-ACTGATGGAAGGGATGTGCA-3′
NM_001038642.2	Ets-1 forward	5′-TCGATCTCAAGCCGACTCTC-3′
	Ets-1 reverse	5′-CATTCACAGCCCACATCACC-3′
NM_001164109.1	Nfatc1 forward	5′-AGATCCCGTTGCTTCCAGAA-3′
	Nfatc1 reverse	5′-TGTGGGATGTGAACTCGGAA-3′
NM_001037177.2	Nfatc2 forward	5′-CCAATCAGTCGGGCTCCTAT-3′
	Nfatc2 reverse	5′-ACCGTTTTCCCAGTGATCCT-3′
NM_001289726.2	GAPDH forward	5′-AGGTCGGTGTGAACGGATTTG-3′
	GAPDH reverse	5′-TGTAGACCATGTAGTTGAGGTCA-3′

## Data Availability

The data that support the findings of this study are available from the corresponding author upon reasonable request.

## Supplementary Materials

The following supporting information can be downloaded at: https://www.mdpi.com/article/10.3390/ijms25094962/s1.
