# Supplementary material for: BAP31 Plays an Essential Role in Mouse B Cell Development via Regulation of BCR Signaling
Source: Int J Mol Sci. 2024 May 2;25(9):4962. doi: 10.3390/ijms25094962 (PMC11084850; doi:10.3390/ijms25094962)
Supplement: Supplementary file 1 [file ijms-25-04962-s001.zip › ijms-2907190-supplementary.pdf]

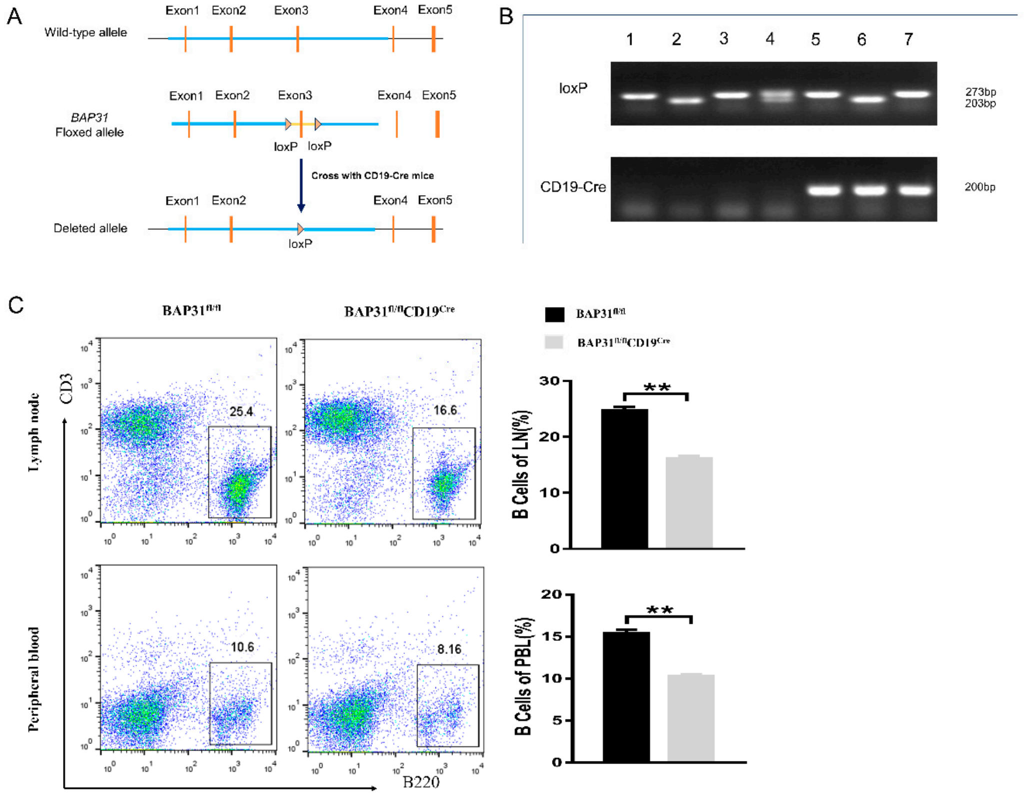

Supplemental Figure S1. (A)Construction strategy of BAP31 conditional knockout mice. Diagram indicating the locations of BAP31 exons, loxP sites and deletion of exon 3 by Cre recombinase. (B) RT-PCR analysis Cre-recombination and mouse genotypes (n=7). Of the seven littermates detected, Lanes 5 and 7 with two bands of 273 bp and 200 bp were identified as BAP31$^{fl/fl}$CD19^{Cre}, Lanes 1 and 3 with one band of 273 bp were identified as BAP31$^{fl/fl}$ mice. (C) Representative flow cytometry profiles of lymph node (LN) and peripheral blood (PBL) from BAP31$^{fl/fl}$ mice and BAP31$^{fl/fl}$CD19^{Cre} mice to identify B cell populations (n=3). Numbers in the plots indicate percentages in each gate.

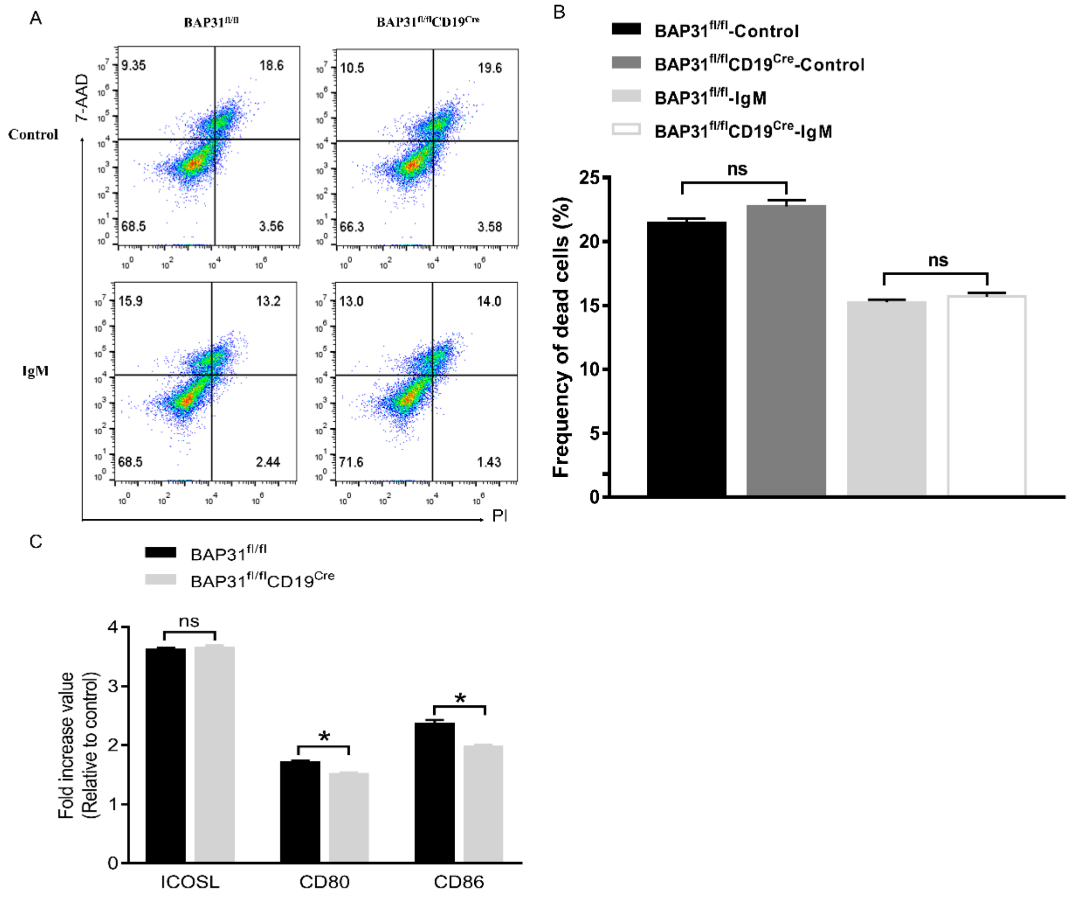

Supplemental Figure S2. (A) Flow cytometry detect the apoptosis of B cells stimulated with IgM using annexin V-FITC/PI staining (n=3). (B) Statistic histogram images of died cells. ns, no significant difference.(C))Fold increase value of ICOSL , CD80, and CD86 compared to corresponding controls (n=3).
